# The Prediction of Drug-Disease Correlation Based on Gene Expression Data

**DOI:** 10.1155/2018/4028473

**Published:** 2018-03-25

**Authors:** Hui Cui, Menghuan Zhang, Qingmin Yang, Xiangyi Li, Michael Liebman, Ying Yu, Lu Xie

**Affiliations:** ^1^School of Life Science and Technology, ShanghaiTech University, Shanghai 201210, China; ^2^Institute for Nutritional Sciences, Shanghai Institutes for Biological Sciences, University of Chinese Academy of Sciences, Shanghai 200031, China; ^3^Shanghai Center for Bioinformation Technology, Shanghai Academy of Science and Technology, Shanghai 201203, China; ^4^College of Food Science and Technology, Shanghai Ocean University, No. 999 Hu Cheng Huan Road, Shanghai 201306, China; ^5^IPQ Analytics, LLC/Strategic Medicine, Philadelphia, PA, USA; ^6^Department of Pharmacology, School of Basic Medical Sciences, Tianjin Medical University, Tianjin 30007, China

## Abstract

The explosive growth of high-throughput experimental methods and resulting data yields both opportunity and challenge for selecting the correct drug to treat both a specific patient and their individual disease. Ideally, it would be useful and efficient if computational approaches could be applied to help achieve optimal drug-patient-disease matching but current efforts have met with limited success. Current approaches have primarily utilized the measureable effect of a specific drug on target tissue or cell lines to identify the potential biological effect of such treatment. While these efforts have met with some level of success, there exists much opportunity for improvement. This specifically follows the observation that, for many diseases in light of actual patient response, there is increasing need for treatment with combinations of drugs rather than single drug therapies. Only a few previous studies have yielded computational approaches for predicting the synergy of drug combinations by analyzing high-throughput molecular datasets. However, these computational approaches focused on the characteristics of the drug itself, without fully accounting for disease factors. Here, we propose an algorithm to specifically predict synergistic effects of drug combinations on various diseases, by integrating the data characteristics of disease-related gene expression profiles with drug-treated gene expression profiles. We have demonstrated utility through its application to transcriptome data, including microarray and RNASeq data, and the drug-disease prediction results were validated using existing publications and drug databases. It is also applicable to other quantitative profiling data such as proteomics data. We also provide an interactive web interface to allow our Prediction of Drug-Disease method to be readily applied to user data. While our studies represent a preliminary exploration of this critical problem, we believe that the algorithm can provide the basis for further refinement towards addressing a large clinical need.

## 1. Introduction

As we know, many diseases are not resolved by treatment with one single drug, for example, most cancers and diabetes. At time of diagnosis and staging, many aberrant genes can be observed, either involving mutation or modification or exhibiting altered levels of expression, yielding perturbations to signaling pathways. This is the reality of complex diseases, which complicates their treatment particularly in the difficulty in identifying potential driver or passenger genes. Therefore, the traditional “one drug-one target” therapeutic approach often shows limited efficacy because of inappropriate targeting, development of adverse events, and potential resistance [1]. As a result, it has become necessary to develop combination drug therapies [2].

Combined drug therapy typically involves administering two or more drugs simultaneously or sequentially. Within the past two decades, combination therapies have been used successfully in clinical experiments and have attracted tremendous attention as promising treatments for complex disorders, especially those with multifactorial pathogenic mechanisms [3]. For example, the combination treatment of fluticasone and propionate provides better asthma control than increasing the dose of either single drug alone, while simultaneously reducing the frequency of exacerbations [4]. It is noted that an increasing number of combination drugs are being marketed as commercial products with a fixed dosage of each component and with approval of the Food and Drug Administration (FDA) in the past 5 years, especially for those complex diseases such as type II diabetes, HIV infections, and cancer. In the particular area of cancer therapy, the first combination was granted in January 2014 by FDA to treat melanoma with BRAF V600E or V600K mutations [2]. Currently, approximately 50 combination therapies, without fixed component dosage, have been referred by FDA to treat different cancer subtypes.

Pharmacologically, a drug combination may produce synergistic, additive, antagonistic, or even suppressive effect if the combined effect is greater than, equal to, or less than the sum of each individual drug [5]. Synergistic effects are typically the most desirable because of enhanced efficacy, potential for decreasing dosage with equal or increased level of efficacy, or delayed development of drug resistance [6]. Therefore, identification of synergistic agents presents a significant opportunity to better deal with complex diseases, even though it is a highly challenging task [7]. The synergy of drugs can be assayed by testing the inhibition of tumor cell growth by individual drugs and their combinations in vitro, followed by a mathematical formulation by Loewe additivity or Bliss independence [1, 8]. However, it is not practical to test the synergistic effect of all possible combinations of drugs through experiments due to the large number of drugs approved by FDA. The development of computational methods for predicting effects of drug combination can play an essential role in developing systematic screening of combinatorial treatment regiments [9].

Previous studies have proposed a handful of computational approaches to analyze high-throughput molecular datasets for predicting the synergy of drug combinations. Recently, Zhao et al. introduced a model to predict the efficacies of drug combinations by integrating molecular and pharmacological data. But its dependence on the feature pattern, specifically enriched in approved drug combinations, severely limited its potential application [10]. Similarly, Wu et al. proposed a network-analysis-based model that utilized gene expression profiles, following individual treatments, to predict gene expression changes induced by drug combinations, which were then used to estimate the effectiveness of the combinations [7]. Another model, named the enhanced Petri-Net model, provided informative insight into the mechanisms of drug actions, which was established to recognize the synergism of drug combinations [11]. But its requirement of a gene expression profile for every drug pair limited its application.

However, these computational approaches only consider the characteristics of the drug itself, without taking into account an equivalent characterization of the disease. The effectiveness of the drug may be applicable for the specified cell line, but not applicable for the actual disease as it presents in patients. To account for this, here we propose an algorithm to specifically predict synergistic effects of drug combinations on various diseases, by integrating the data characteristics of disease-related gene expression profiles with drug-treated gene expression profiles. We have demonstrated utility through its application to transcriptome data, including microarray and RNASeq data, and the drug-disease prediction results were validated using existing publications and drug databases. It is also applicable to other quantitative profiling data such as proteomics data. We also provide an interactive web interface (https://www.scbit.org/PEDD/) to allow our Prediction of Drug-Disease method to be readily applied to user data.

## 2. Methods

In this research, we developed a disease-drug prediction algorithm using transcriptome data. We describe both data aggregation and our algorithm in detail, below.

### 2.1. Data Aggregation

First, gene expression data of drug treated samples and disease-related gene expression dataset are identified and qualified from literature and public domain databases.

#### 2.1.1. Gene Expression Data following Drug Treatment

GSE51068 dataset (https://www.ncbi.nlm.nih.gov/geo/query/acc.cgi?acc=GSE51068) was downloaded from the GEO database, which contained gene expression data of 282 drug-treated samples. We selected high-throughput expression profiling of OCI-Ly3 cell line treated with 14 different known drugs at 2 different concentrations and profiled at 6, 12, and 24 hours after treatment. For our initial study, profiling after 6-hour treatment was chosen. Summary information about the 14 known drugs was shown in Table S1.

#### 2.1.2. Disease-Related Gene Expression Data

We have developed our method so that it can be applied not only to microarray data, but also to RNAseq data. Thus, two data types were identified and collected.

We established the following requirements for microarray data in this study: the experimental group involves human disease samples; the control group is nondisease samples; and the number of experimental samples is greater than 50. Six microarray datasets (GSE9476, GSE33615, GSE22529, GSE26049, GSE19429, and GSE47552) were selected from the GEO database (https://www.ncbi.nlm.nih.gov/geo/), including 9 blood cell and bone marrow related malignancies and diseases (Table S2).

Additional disease-related gene expression data involves RNAseq data. Here, four cancer types were chosen including breast cancer, liver cancer, lung adenocarcinoma, and lung squamous cell carcinoma. We extracted these cancer-related RNAseq data from UCSC Xena, which is provided by TCGA (https://xenabrowser.net/datapages/?host=https://tcga.xenahubs.net).

### 2.2. Algorithm Design and Implementation

**Figure 1:**
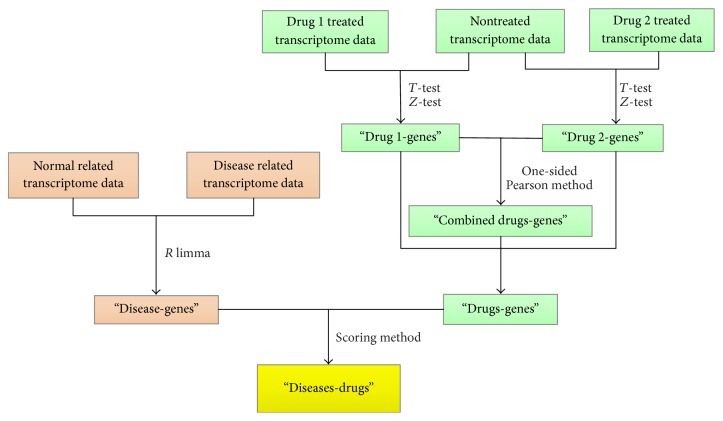
The algorithm flow.

Our goal is to predict the effects of drugs on various diseases when used in combination. The detailed algorithm implementation is defined in the steps (Figure 1).

**Step 1 .**

Differentially expressed genes (DEGs) were identified within the disease-related gene expression dataset. For microarray data, the “limma” package in *R* was used to identify DEGs, with a Benjamini-Hochberg adjusted *p* value of 0.01. For RNASeq data, the “limma” package in *R* was also used to identify DEGs, with a Benjamini-Hochberg adjusted *p* value of 0.05. Additionally, the threshold fold change in gene expression in the experimental group that was selected was at least twice higher or lower than the gene expression in control group for microarray and RNASeq data.

**Step 2 .**

DEGs were identified for the 14 drugs. A *T* test was performed to get the observed test statistics for the genes in the drug-treated group compared to control group. Then, the observed test statistics were converted into *z*-scores:

(1) $$\begin{array}{c} z_{i}=\Phi^{-1}\left(\;P\left(\;t_{i}\;\right)\;\right), \end{array}$$

where *t*_*i*_ denotes the observed test statistics for the gene *i* and Φ(·) is the cumulative distribution. If the *z*-score is greater than 1.96, it indicates that the gene expression is upregulated after drug treatment. If the *z*-score is lower than −1.96, it indicates that the gene expression is downregulated after drug treatment.

**Step 3 .**

DEGs were identified for the 91 combination drugs. The 14 drugs will generate 91 unique drug combinations (*C*_14_^2^). To compute the combined effect of two drugs on each gene, a one-sided Pearson's method was used to combine the *z*-scores of two drugs:

(2) $$\begin{array}{c} p_{i}^{s}=P\left(\;X_{4}^{2}<-2\times \sum_{j=1,2}\ln\left(\;1-\Phi \left(\;z_{ij}\;\right)\;\right)\;\right), \end{array}$$

where *z*_*ij*_  (*j* = 1,2) denote the *z*-score of the gene *i* for any two drugs.

Then, the combined *z*-score was calculated:

(3) $$\begin{array}{c} z_{i}^{\prime }=\Phi^{-1}\left(\;p_{i}^{s}\;\right). \end{array}$$

**Step 4 .**

DEGs of drug-related and disease-related were matched by evaluating a specific constraint. Here, the *p* value of the “drug-disease” relationship is calculated using the following formula:

(4) $$\begin{array}{c} p^{k}=\Phi \left(\;\frac{\sum_{i=1}^{n}abs\left(\Phi^{-1}\left(p_{i}^{s}\right)\right)I\;\;\left\{i\in k\right\}}{\sqrt{\sum_{i=1}^{k}abs\left(I\right)\;\;\left\{i\in k\right\}}}\;\right), \end{array}$$

where *k* represents the number of genes that can be matched between the drug and the disease and *I* is the matching coefficient (Table 1). If the gene is upregulated in the disease and the gene is downregulated after drug treated, *I* is +1. If the gene is downregulated in the disease and the gene is upregulated after drug treated, *I* is +1. Otherwise, *I* is −1.

**Step 5 .**

An indicator score was calculated, by scoring the matching results, to evaluate the effect of the drug on the disease. The formula is as follows:

(5) $$\begin{array}{c} Score=\frac{\Phi^{-1}\left(P^{k}\right)\times k}{N}, \end{array}$$

where *k* represents the number of genes that can be matched between the drug and the disease. *N* is the total number of DEGs in each disease. *P* is the value calculated in Step 4.

### 2.3. Literature and Database Validation

For any two drugs (A and B) and any specific disease, three scores can be generated, indicating the relationship between drug A and the disease, between drug B and the disease, and between the A + B drug combination and the disease. Here, we chose the highest score as the most effective. In addition, the score must be greater than 0, suggesting that the drug has an enhanced treatment effect on the disease. If the score of drug combination is higher than that of any single drug, we define the drug combination to be more effective. We chose to exclude those drugs that were not in DrugBank. Finally, results were validated through reviewing both published literature and drug-related databases, including DrugBank (https://www.drugbank.ca/releases/latest) [12], FDA (https://www.fda.Gov/), DCDB (http://www.cls.zju.edu.cn/dcdb/) [13], and the Pubmed (https://www.ncbi.nlm.nih.gov/pubmed).

## 3. Results

### 3.1. Relation between Drug and Disease

**Figure 2:**
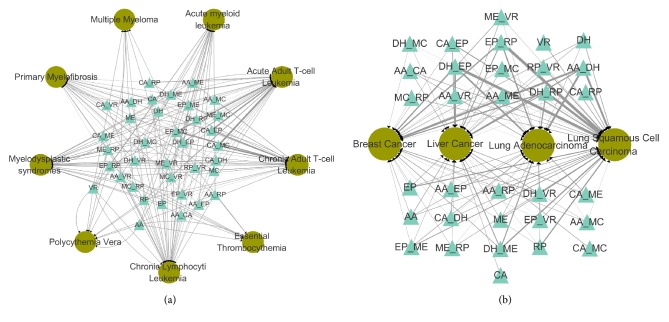
The relationship between drug and disease using microarray data (a) and RNASeq data (b). Drugs are represented by triangles. Diseases are represented by circles. The thickness of the linking edge is directly related to the magnitude of the score between drug and disease.

As a result of our analysis, relationships between drugs and diseases were established and are shown in Figure 2(a) for microarray data. We can see that the most closely related to acute adult T-cell leukemia is the drug combination of camptothecin (CA) and Mitomycin C (MC), followed by the drug combination of camptothecin (CA) and Etoposide (EP) and combination of Etoposide (EP) and Mitomycin C (MC). These drug combinations were also closely related to chronic adult T-cell leukemia, which may be due to their similar pathophysiologic characteristics.

Similarly, relationships between drugs and other cancers are shown in Figure 2(b) for RNASeq data. The drug combination most closely related to breast cancer is that of Aclacinomycin A (AA) and Doxorubicin (DH), followed by the drug combination of Doxorubicin (DH) and Etoposide (EP) and then the combination of Etoposide (EP) and Rapamycin (RP). The most closely related combination to liver cancer involves Doxorubicin (DH) and Etoposide (EP), followed by the drug combination of Aclacinomycin A (AA) and Doxorubicin (DH) and then the combination of Etoposide (EP) and Rapamycin (RP). The drug combination most closely related to lung adenocarcinoma is Aclacinomycin A (AA) and Doxorubicin (DH), followed by the drug combination of Doxorubicin (DH) and Etoposide (EP) and then the combination of Doxorubicin (DH) and Rapamycin (RP). In lung squamous cell carcinoma the most closely related drug combination involves Etoposide (EP) and Rapamycin (RP), followed by the drug combination of Doxorubicin (DH) and Etoposide (EP) and then the combination of Doxorubicin (DH) and Rapamycin (RP).

### 3.2. Further Validation

As a result of our filtering algorithm (see Methods), a total of 105 relationships between drugs and diseases were identified using microarray data, and a total of 67 relationships were identified using RNASeq data. Then, results were validated through review of published literature and drug-related databases.

The reviewing identified 36 relationships (microarray) and 41 relationships (RNASeq) in previous studies (Tables S3 and S4). Moreover, there are also 39 synergistic drugs and 18 synergistic drugs identified by previous studies, for microarray and RNASeq data, respectively (Tables S5 and S6).

### 3.3. Web Interface

We have further implemented the proposed approach as an interactive web tool, named “Predicting the Effect of the Drug on Disease (PEDD)” (https://www.scbit.org/PEDD/). This web tool is intuitive and can be easily applied to similar analyses using user-provided drug-treated gene expression data and disease-related gene expression data, to predict relationships between drugs and diseases. We continue to refine the algorithm and to refine the selection of datasets, for example, both experimental data and disease subtypes, in ongoing studies.

## 4. Discussion

Due to the complexity of the disease, frequent lack of response to targeted therapies, and the emergence of drug resistance, interest in potential drug combination therapy has increased [14]. Both computational methods and experimental methods have been applied to screen synergistic drugs. An optimal approach would be the potential to use computational screening to broaden the study of potential component drugs for combination therapy and to better direct the application of experimental validation. This approach can lead to more rapid and effective means for screening and identifying candidate drug combinations. Synergistic drug prediction models have been previously studied. For example, Jin et al. built an enhanced Petri-net (EPN) model to predict the synergistic effect of pairwise drug combinations from genome-wide transcriptional expression data, by applying Petri-nets to identify specific drug targeted signaling networks [11]; Sun et al. constructed a model called Ranking-system of Anticancer Synergy (RACS) based on semisupervised learning which was used to rank drug pairs according to their similarity to the labeled samples in a specified multifeature space [15]. However, these computational approaches only considered the characteristics of the drug itself, without taking into account potentially valuable disease observations. The resulting effectiveness of these predictions may be applicable for the cell line, but not readily extendable for disease as it appears in humans. For these reasons, we developed an algorithm to expand on these earlier works and to predict the effects of drugs on various diseases, by integrating gene expression data generated from disease tissues and drug-treated cell lines.

The workflow is as follows. Firstly, up and down genes were calculated with disease-related gene expression data. Secondly, with the gene expression data of drug-treated cell line, we calculated up and down genes for single drug and combination drugs. Next, the disease-related up and down genes were matched with drug-related up and down genes by our matched principle. Moreover, according to the matched result, scores were calculated which represented the effect of drug on various diseases by our scoring method. The implementation of our algorithm as an interactive web tool makes the proposed approach easily accessible to all scientists in general. Researchers can find potential drugs for diseases according to the calculated scores.

In this study, our algorithm can give out the scores of both drug combination and each of the single drug for a disease; thus it is applicable not only to the drug combination prediction, but also to the drug repositioning. Also, according to the score rank, it may be defined that the drug combination is more effective than single drugs if it has the highest score. Besides, this algorithm is not only applicable to transcriptomics data, but also applicable to other quantitative profiling data, such as proteomics data.

The results showed that the effect of combination drugs may be higher than the effect of the individual component drugs in some diseases. For example, the effect of combination of camptothecin and monastrol was predicted to be greater than the effect of camptothecin or monastrol, individually, in acute adult T-cell leukemia and chronic adult T-cell leukemia. In contrast, the effect of combination drugs may be lower than the effect of the individual component drugs in some other diseases. For example, the effect of combination of camptothecin and monastrol was predicted to be reduced in efficacy in multiple myeloma and polycythemia vera. In general, we believe that this analytic approach can contribute to drug research and screening studies and use this preliminary study to show its potential value.

However, in our algorithm, differential genes bear equal weights while the change of some key genes may give larger effect. For example, both gene sequence variations and expression changes are important molecular phenotypes in human disease, especially cancer. They should be assigned differential weights. But, how to determine the key genes and how to assign differential weights for them are very difficult, as we only use the data of gene expression profile in this study. In the future research, more in-depth study of this aspect considering more factors should be carried out. For example, we may use multilevel omics expression data and drug targets to find the key genes and assign differential weights for them. What is more, we also recognize that the disease classes, for example, “breast cancer,” that have been used in this study are likely subject to further stratification, for example, DCIS. We are currently studying the application of this approach to such refinements.

And with the rapid development of next-generation sequencing (NGS) technology and the accumulation of histological data [16], there have been many databases that can be used to screen single drugs or synergistic drugs such as FDA and DrugBank [12]. However, a comprehensive database about “drug-cancer relationships” has not been established, which contains both the single drugs and combination drugs related to cancer-related information. We believe such database would be available in future, by collecting the information from current public databases and published literature. The database will provide an important assessment criteria for the “drug-cancer” predictions and provide important reference value for the strategy design of antitumor combination therapy. While our studies represent a preliminary exploration of this critical direction, we believe that the algorithm can provide the basis for further refinement towards addressing a large clinical need in antitumor combination therapy.

## Figures and Tables

**Table 1:** The matching coefficient.

Disease	Drug	
	Up expressed gene	Down expressed gene
Up expressed gene	−1	+1
Down expressed gene	+1	−1

## Supplementary Materials

Supplementary Materials Table S1: drug information. Table S2: microarray data information. Table S3: drug-disease relations identified by previous studies from microarray data. Table S4: drug-disease relations identified by previous studies from RNAseq data. Table S5: synergistic drugs identified by previous studies from microarray data. Table S6: synergistic drugs identified by previous studies from RNAseq data.
