# Supplementary material for: The Prediction of Drug-Disease Correlation Based on Gene Expression Data
Source: Biomed Res Int. 2018 Mar 25;2018:4028473. doi: 10.1155/2018/4028473 (PMC5889901; doi:10.1155/2018/4028473)
Supplement: Supplementary Materials — Table S1: drug information. Table S2: microarray data information. Table S3: drug-disease relations identified by previous studies from microarray data. Table S4: drug-disease relations identified by previous studies from RNAseq data. Table S5: synergistic drugs identified by previous studies from microarray data. Table S6: synergistic drugs identified by previous studies from RNAseq data. [file 4028473.f1.docx]

Table S1: Drug information.

| Drug Name (Abbreviation) | Category | Application | DrugBank ID |
| --- | --- | --- | --- |
| Aclacinomycin A (AA) | Antibiotic | - | DB11617 |
| Camptothecin (CA) | Antineoplastic enzyme inhibitor | Metastatic colorectal cancer | DB00762 |
| Doxorubicin hydrochloride (DH) | Cytotoxic anthracycline antibiotic | Disseminated neoplastic | DB00997 |
| Etoposide (EP) | Cell cycle specific agents | Anti-cancer | DB00773 |
| Mitomycin C (MC) | Antineoplastic antibiotic | Malignant neoplasm | DB00305 |
| Methotrexate (ME) | Antineoplastic antimetabolite with immunosuppressant properties | Gestational choriocarcinoma, chorioadenoma destruens and hydatidiform mole | DB00563 |
| Rapamycin (RP) | Antifungal and immunosuppressive agents | The prophylaxis of organ rejection | DB00877 |
| Vincristine (VR) | Antitumor vinca alkaloid isolated | Anti-tumor | DB00541 |
| Blebbistatin (BL) | Cell permeability inhibitor | - | - |
| Cycloheximide (CY) | Protein biosynthesis inhibitor | - | - |
| Geldanamycin (GD) | HSP90 inhibitor | - | - |
| Trichostatin A (TA) | HDAC inhibitors | - | - |
| H-7, Dihydrochloride (HD) | Chemical substances | - | - |
| Monastrol (MA) | Chemical substances | - | - |

Table S2: Microarray data

| GEO Data Set | Disease Name | Abbreviation |
| --- | --- | --- |
| GSE9476 | Acute myeloid leukemia | AML |
| GSE22529 | Chronic Lymphocyti Leukemia | CLL |
| GSE33615 | Acute Adult T-cell Leukemia | Acute_ATL |
|  | Chronic Adult T-cell Leukemia | Chronic_ATL |
| GSE47552 | Multiple Myeloma | MM |
| GSE19429 | Myelodysplastic syndromes | MDS |
| GSE26049 | Primary Myelofibrosis | PMF |
|  | Polycythemia Vera | PV |
|  | Essential Thrombocythemia | ET |

Table S3: Drug-disease relations identified by previous studies from microarray data.

| Drug | Disease | Score | Database(ID) | Pubmed |
| --- | --- | --- | --- | --- |
| CA_MC | Acute_ATL | 102.9497 | - | 10854134 |
| CA_MC | Chronic_ATL | 92.06085 | - | 10854134 |
| EP_MC | Acute_ATL | 74.8417 | - | 11724325 |
| EP_MC | Chronic_ATL | 70.69611 | - | 11724325 |
| EP_RP | Acute_ATL | 44.39987 | - | 26886430 |
| DH_ME | Acute_ATL | 37.38928 | - | 25066676 |
| EP_RP | Chronic_ATL | 37.20129 | - | 26886430 |
| EP_ME | Acute_ATL | 36.68179 | - | 4075296 |
| DH_MC | Chronic_ATL | 32.44924 | DCDB(DC000408) |  |
| EP_RP | PMF | 30.23127 | - | 26886430 |
| EP_ME | Chronic_ATL | 29.81993 | - | 4075296 |
| DH_MC | Acute_ATL | 28.86069 | DCDB(DC000408) |  |
| AA_DH | Acute_ATL | 28.42668 | - | 2803945 |
| CA_ME | Acute_ATL | 26.67396 | - | 1537625 |
| DH_ME | Chronic_ATL | 18.21455 | - | 25066676 |
| EP | Chronic_ATL | 16.29341 | Drugbank(DB00773) | - |
| AA_VR | Acute_ATL | 12.29815 | - | 2643396 |
| EP | Acute_ATL | 11.59762 | Drugbank(DB00773) | - |
| ME_VR | Acute_ATL | 11.56089 | - | 526921 |
| AA_DH | Chronic_ATL | 11.00588 | - | 2803945 |
| ME | Acute_ATL | 7.05083 | Drugbank(DB00563) |  |
| DH_RP | CLL | 6.886392 | - | 25482130 |
| ME_VR | Chronic_ATL | 5.40395 | - | 526921 |
| RP | MM | 3.843232 | Drugbank(DB06287) | - |
| AA_VR | AML | 2.938843 | - | 2643396 |
| ME | Chronic_ATL | 1.925906 | Drugbank(DB00563) | - |
| DH_MC | MDS | 1.924734 | DCDB(DC000408) | - |
| EP_VR | CLL | 1.565152 | DCDB(DC000581) | - |
| EP_MC | CLL | 1.432344 | - | 11724325 |
| ME_VR | CLL | 1.225539 | - | 526921 |
| DH_EP | AML | 0.86524 | - | 18488159 |
| AA | AML | 0.693563 | - | 6449134 |
| EP | AML | 0.686212 | Drugbank(DB00773) |  |
| EP_RP | MDS | 0.537242 | - | 26886430 |
| EP | CLL | 0.512805 | Drugbank(DB00773) |  |
| DH_MC | AML | 0.249026 | DCDB(DC000408) |  |

Table S4: Drug-disease relations identified by previous studies from RNAseq data

| Drug | Disease | Score | Database(ID) | Pubmed |
| --- | --- | --- | --- | --- |
| EP_RP | LUSC | 110.1432 | - | 26886430 |
| DH_EP | LUSC | 106.7887 | - | 20416058 |
| DH_EP | LUAD | 72.00459 | - | 20416058 |
| DH_EP | BRAC | 62.0341 | - | 20951586 |
| EP_RP | LUAD | 61.83473 | - | 26886430 |
| EP_RP | BRAC | 61.19565 | - | 26886430 |
| DH_ME | LUSC | 60.07273 | - | 8394198 |
| DH_RP | BRAC | 59.41896 | - | 24726747 |
| RP | LUSC | 58.44756 | - | 24658085 |
| DH_ME | LUAD | 53.93909 | - | 8394198 |
| EP | LUSC | 53.35931 | Drugbank(DB00773) | - |
| EP_RP | LIHC | 51.59021 | - | 26886430 |
| DH_VR | LUSC | 51.29165 | DCDB(DC003898) | 10080612 |
| DH_VR | LUAD | 49.55222 | DCDB(DC003898) | 10080612 |
| DH_VR | BRAC | 42.26461 | DCDB(DC003898) | 6344982 |
| DH | LUSC | 40.88441 | DCDB(DCC0259) | - |
| DH_VR | LIHC | 40.60098 | DCDB(DC003898) | - |
| EP_ME | LUAD | 39.40966 | - | 8384659 |
| DH | BRAC | 37.98929 | DCDB(DCC0259) | - |
| EP | LIHC | 37.1197 | Drugbank(DB00773) | - |
| DH | LIHC | 36.17961 | DCDB(DCC0259) | - |
| EP | LUAD | 35.21697 | Drugbank(DB00773) | - |
| DH | LUAD | 34.12397 | DCDB(DCC0259) | - |
| RP | BRAC | 31.29089 | - | 25605156 |
| EP | BRAC | 29.93802 | Drugbank(DB00773) | - |
| RP | LUAD | 29.25703 | - | 24658085 |
| AA_MC | LUAD | 19.84879 | - | 3820605 |
| AA_MC | LUSC | 19.09801 | - | 3820605 |
| AA_VR | LUAD | 17.33207 | - | 26021434 |
| ME_VR | LUAD | 14.98827 | - | 8384659 |
| ME_VR | LUSC | 14.91263 | - | 8384659 |
| AA | BRAC | 9.30655 | - | 6596963 |
| ME | LIHC | 9.138159 | - | 9428374 |
| CA | LIHC | 8.905348 | - | 24861663 |
| AA_VR | LUSC | 8.831095 | - | 26021434 |
| ME | LUAD | 7.312838 | FDA | - |
| AA | LUAD | 6.937843 | FDA | 2574988 |
| AA | LUSC | 5.804537 | FDA | 2574988 |
| ME | LUSC | 3.198007 | FDA | - |
| AA | LIHC | 3.10629 | - | 2846313 |
| VR | LUSC | 0.251173 | - | 2167702 |

Table S5: Synergistic drugs identified by previous studies from microarray data

| Drug | Disease | Score | Database(ID) | Pubmed ID |
| --- | --- | --- | --- | --- |
| CA_EP | Acute_ATL | 78.52702 | - | colon carcinoma,1326304 |
| CA_EP | Chronic_ATL | 74.6178 | - | colon carcinoma,1326304 |
| DH_EP | Acute_ATL | 58.85077 | - | acute myelogenous leukemia,  18488159 |
| DH_EP | Chronic_ATL | 54.80303 | - | acute myelogenous leukemia,  18488159 |
| CA_RP | Acute_ATL | 52.89196 | - | brain tumor,11245461 |
| CA_RP | Chronic_ATL | 44.87901 | - | brain tumor,11245461 |
| CA_DH | Chronic_ATL | 40.28331 | - | Oral cancer，25734832,  breast cancer,25921087 |
| CA_DH | Acute_ATL | 34.70646 | - | Oral cancer，25734832,  breast cancer,25921087 |
| ME_MC | Acute_ATL | 31.76469 | - | breast cancer,6401590 |
| DH_EP | PMF | 28.90826 | - | acute myelogenous leukemia,  18488159 |
| EP_MC | PMF | 26.11021 | - | leukemia,11724325 |
| ME_MC | Chronic_ATL | 25.16144 | - | breast cancer,6401590 |
| CA_EP | PMF | 23.51929 | - | colon carcinoma,1326304 |
| DH_RP | PMF | 16.29156 | - | T-cell acute lymphoblastic leukemia,  25482130 |
| ME_RP | Acute_ATL | 16.19937 | - | osteosarcoma,26924291 |
| AA_MC | Acute_ATL | 15.29224 | - | cervical adenocarcinoma，15228422,  gastric cancer，2105085；  cervical adenocarcinoma,10870306 |
| AA_ME | Acute_ATL | 15.29224 | - | Ewing's sarcoma,3478002 |
| CA_RP | CLL | 14.76376 | - | brain tumor,11245461 |
| CA_RP | PMF | 12.81052 | - | brain tumor,11245461 |
| CA_DH | PMF | 12.49293 | - | Oral cancer,25734832；  breast cancer,25921087； |
| CA_MC | PMF | 10.99555 | - | leukaemia,10854134 |
| CA_DH | CLL | 9.873212 | - | Oral cancer,25734832；  breast cancer,25921087； |
| ME_RP | Chronic_ATL | 5.30718 | - | osteosarcoma,26924291 |
| ME_MC | PMF | 4.607642 | - | breast cancer,6401590 |
| DH_EP | CLL | 4.585796 | - | acute myelogenous leukemia,  18488159 |
| RP_VR | MM | 4.277468 | - | acute heart allograft rejection,  12740883 |
| MC_VR | MM | 2.217423 | - | cervical squamous carcinoma,  26107215 |
| RP_VR | ET | 2.167785 | - | acute heart allograft rejection,  12740883 |
| AA_VR | ET | 2.090123 | - | non-Hodgkin's lymphoma,2029185 |
| MC_VR | AML | 1.893903 | - | cervical squamous carcinoma,  26107215 |
| DH_ME | MDS | 1.801848 | - | leukaemia,25066676 |
| RP_VR | PV | 1.787775 | - | acute heart allograft rejection,  12740883 |
| AA_VR | CLL | 1.48877 | - | non-Hodgkin's lymphoma,2029185 |
| ME_RP | MDS | 1.191965 | - | osteosarcoma,26924291 |
| EP_MC | MDS | 1.131466 | - | leukemia,11724325 |
| AA_VR | PV | 0.790077 | - | non-Hodgkin's lymphoma，2029185 |
| ME_VR | MDS | 0.416452 | - | leukemia,526921 |
| AA_VR | Chronic_ATL | 0.362518 | - | non-Hodgkin's lymphoma，2029185 |
| AA_ME | MDS | 0.174289 | - | Ewing's sarcoma,3478002 |
| AA_VR | MDS | 0.013228 | - | non-Hodgkin's lymphoma,2029185 |

Table S6: Synergistic drugs identified by previous studies from RNAseq data.

| Drug | Disease | Score | Database(ID) | Pubmed |
| --- | --- | --- | --- | --- |
| DH_RP | LUSC | 97.71421 | - | BRAC,24726747 |
| AA_DH | LUSC | 83.36217 | - | leukemia,2803945 |
| AA_DH | LUAD | 80.90045 | - | leukemia,2803945 |
| AA_DH | BRAC | 80.75019 | - | leukemia,2803945 |
| DH_EP | LIHC | 68.54683 | - | acute myelogenous leukemia,18488159 |
| AA_DH | LIHC | 57.57861 | - | leukemia,2803945 |
| DH_RP | LUAD | 57.25951 | - | BRAC,24726747 |
| DH_RP | LIHC | 51.2805 | - | BRAC,24726747 |
| CA_EP | LIHC | 45.22961 | - | rat glioma cells,11592782 |
| CA_DH | LIHC | 43.28909 | - | BRAC,25921087 |
| AA_EP | BRAC | 33.09018 | - | acute myelogenous leukemia,3162695 |
| MC_RP | LIHC | 25.38267 | - | pancreatic cancer,25505613 |
| AA_ME | LUAD | 19.84879 | - | Ewing's sarcoma,3478002 |
| AA_ME | LUSC | 19.09801 | - | Ewing's sarcoma,3478002 |
| AA_VR | BRAC | 15.40374 | - | BRAC,26021434 |
| AA_ME | LIHC | 13.84452 | - | Ewing's sarcoma,3478002 |
| AA_ME | BRAC | 10.01619 | - | Ewing's sarcoma,3478002 |
| AA_VR | LIHC | 3.880201 | - | BRAC,26021434 |
